# Combination of compressed sensing-based iterative reconstruction and offset acquisition for I-123 FP-CIT SPECT: a simulation study

**DOI:** 10.22038/AOJNMB.2021.59585.1417

**Published:** 2022

**Authors:** Norikazu Matsutomo, Takeyuki Hashimoto, Mitsuha Fukami, Tomoaki Yamamoto

**Affiliations:** Department of Medical Radiological Technology, Faculty of Health Sciences, Kyorin University, Tokyo, Japan

**Keywords:** Compressed sensing, Rapid acquisition, Dynamic SPECT, Dopamine transporter imaging, SPECT reconstruction

## Abstract

**Objective(s):**

The purpose of this study was to validate undersampled single-photon emission computed tomography (SPECT) imaging using a combination of compressed sensing (CS) iterative reconstruction (CS-IR) and offset acquisition.

**Methods:**

Three types of numerical phantoms were used to evaluate image quality and quantification derived from CS with offset acquisition. SPECT images were reconstructed using filtered back-projection (FBP), maximum likelihood-expectation maximization (ML-EM), CS-IR, and CS-IR with offset acquisition. The efficacy of CS-IR with offset acquisition was examined in terms of spatial resolution, aspect ratio (ASR), activity concentration linearity, contrast, percent coefficient of variation (%CV), and specific binding ratio (SBR).

**Results:**

The full widths at half maximum remained unchanged as the number of projections decreased in CS-IR with offset acquisition. Changes in ASRs and linearities of count density were observed for ML-EM and CS-IR from undersampled projections. The %CV obtained by CS-IR with offset acquisition was substantially lower than that obtained by ML-EM and CS-IR. There were no significant differences between the %CVs obtained from 60 projections by CS-IR with offset acquisition and from 120 projections by FBP. Although the SBRs for CS-IR with offset acquisition tended to be slightly lower than for FBP, the SBRs for CS-IR with offset acquisition did not change with the number of projections.

**Conclusions:**

CS-IR with offset acquisition can provide good image quality and quantification compared with a commonly used SPECT reconstruction method, especially from undersampled projection data. Our proposed method could shorten overall SPECT acquisition times, which would benefit patients and enable quantification with dynamic SPECT acquisitions.

## Introduction

 Single-photon emission computed tomo-graphy (SPECT) images are typically recon-structed by acquiring projection data from multiple different angles around the patient. Projection data, which are obtained by rotating the gamma camera, are required from 64 to 128 views based on the sampling theorem (1). In several imaging guidelines, the optimal projection number is recommended to be 120 views for dopamine transporter imaging with ^123^I-ioflupane SPECT (2, 3) and 64 or 128 views over 180° or 128 views over 360° for myocardial SPECT imaging using ^99m^Tc (4, 5). Therefore, the acquisition of projection data with adequate image quality is time-consuming, which is a practical problem for clinical SPECT imaging because the patient needs to remain still for a long period of time.

 Compressed sensing (CS) is a signal processing technique that was initially proposed for sparse signal recovery, and CS iterative reconstruction (CS-IR) can be performed from fewer data than typical images 

(6, 7). In CS-IR algorithm, the total variation (TV) is a regularization method to minimized the TV norm which is the L1 norm of the gradient of the image, the TV norm has the ability to suppress small changes in the value of a pixel in response to the value of surrounding pixels (large changes remain). This effect essentially suppresses streaking artifacts and noise in the reconstructed image. We have reported the use of CS-IR with undersampled projection data to shorten acquisition time (8). We assessed the effect of CS-IR on ^123^I-ioflupane SPECT from undersampled projection data. CS-IR reduced the projection number and total acquisition time by approximately two-thirds without decreasing the image quality and quantification. However, CS-IR was not appropriate for very small projection numbers because, while it can mitigate the effects of streak artifacts and projection numbers, the reconstructed images exhibit a specific patchy pattern behavior. Therefore, further reductions in the projection number and the total acquisition time require the introduction of different characteristics to the CS-IR algorithm.

**Figure 1:**
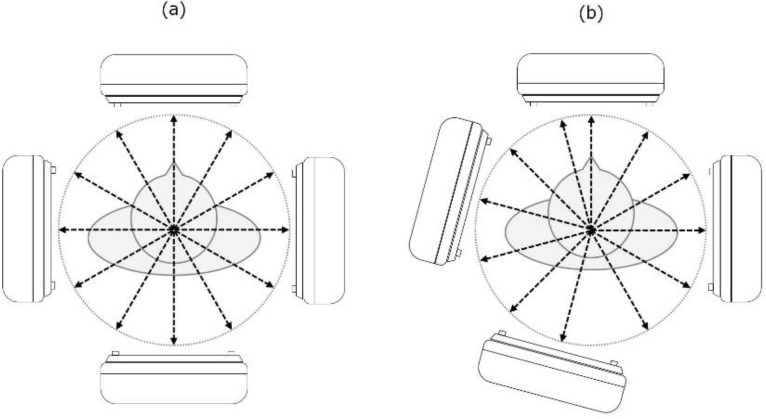
Schematic of the offset acquisition method. (a) Conventional single-photon emission computed tomography (SPECT) acquisition using a dual-head SPECT system and (b) offset acquisition. Projection data generated by the offset acquisition are asymmetric because the opposite projection data are generated by offsetting opposite gamma cameras by half the step angle

 Takahashi et al. developed wide-angle offset acquisition to reduce the projection number and the total acquisition time (9). Offset acquisition is an asymmetric projection data method in which opposite gamma cameras are shifted by half the step angle (Figure 1). This technique improves the spatial resolution of the SPECT image and reduces the total acquisition time compared with the current SPECT acquisition method. A combination of the advantages of CS-IR and offset acquisition may enable image reconstruction with a smaller projection number. This technique would help to reduce the constrained time and patient motion artifacts because lowering the projection number is a simple way to reduce the SPECT acquisition time.

 In this study, we achieved a shorter acquisition time from undersampled SPECT imaging data using a combination of our CS-IR method and offset acquisition. We evaluated the quality and quantification of the SPECT imaging data obtained from undersampled projection data by CS-IR with offset acquisition (CS-IR with offset) using digital mathematical and striatal numerical phantoms.

## Methods

***Phantom design***

**Figure 2:**
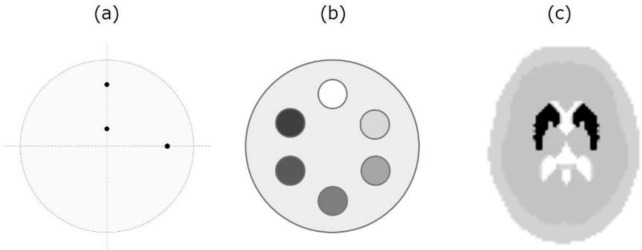
Simulation phantom configuration. (a) Resolution phantom, (b) multi-cylinder phantom, and (c) three-dimensional striatum digital brain phantom. The multi-cylinder phantom is 160 mm in diameter, 150 mm tall, and contains six cylinders. The striatum-to-brain tissue ratios in the striatal phantom were set as 8 (striatum)

 Three types of numerical phantoms were used to evaluate image quality and quantification (Figure 2). Spatial resolution was evaluated using a cylinder phantom consisting of three point sources set at 10 and 100 mm (upper and right) from the center of the cylinder (Figure 2a). Point source and background counts were set at 800 and 10 counts/pixel, respectively. The linearity of the count density was assessed using a multi-cylinder phantom composed of six cylinders with a diameter of 40 mm. The background count was set at 100 counts/pixel, and the cylinder counts were assigned as 0, 200, 300, 400, 600, and 800 counts/pixel (Figure 2b). To investigate the uniformity and quantification, a three-dimensional striatum digital brain (3D-SDB) phantom (10) was used as an anthropomorphic phantom. The 3D-SDB phantom consisted of four segments (the striatum, ventricle, brain parenchyma, and bone). Image structures were extracted from T1- and T2-weighted images. The striatum count and the brain tissue count were 800 and 100 counts/pixel, respectively. The skull bone and ventricle counts were 20 and 0 counts/pixel, respectively (Figure 2c).

***Simulation conditions***

 Simulating Medical Imaging Nuclear Detectors (SIMIND) Monte Carlo program version 6.2 was used to generate the projection data (11, 12). The simulated SPECT system was a dual-head SPECT/CT camera (Infinia Hawkeye 4, GE Healthcare, Chicago, IL) equipped with a low-energy, high-resolution parallel-hole collimator. The projection data for each phantom were generated in 128×128 matrices with a 3.2×3.2 mm pixel and 360° clockwise gantry rotation. The rotation radius was set at 150 mm for the point, multi-cylinder, and 3D-SDB phantoms. The simulated count was approximately 75 kilo-counts per projection, and additive Poisson statistical noise was randomly set for each projection. In this study, we evaluated the performance of CS-IR and CS-IR with offset for undersampled projection data. Therefore, the projection number was varied from 8 to 120 projections after the projection data were generated from 360 projections. Because the acquisition time for each projection was kept constant, the total acquisition time was reduced to approximately 1/15 as the number of projection data changed from 120 to 8 projections. In current SPECT acquisition, the projection data are separated by a constant angular interval. In offset acquisition, the opposite projection data are generated by offsetting opposite gamma cameras by half the step angle; that is, for 60 projections, the projection data derived from detector 1 are in the range 0° to 174° and the projection data derived from detector 2 are in the range 183° to 357°. Therefore, the apparent projection number (asymmetric projection data) is increased in the offset acquisition method.

***Image reconstruction***

 Image reconstruction from onset acquisition (current SPECT acquisition) was performed by maximum likelihood-expectation maximization (ML-EM) and CS-IR (6, 13). In addition, simulated offset projection data were reconstructed by the CS-IR with offset. The TV was used for the sparse transformation in the iterative process. The pixels, fi, in the 2D transverse slice image of an image to be reconstructed are updated iteratively by

$f_{i}^{\mathrm{new}}=\frac{f_{i}^{\mathrm{old}}}{\sum_{j}C_{\mathrm{ij}}\left\{1+\beta \frac{\partial }{\partial f_{i}}U_{\mathrm{TV}}\left(f^{\mathrm{old}}\right)\right\}}\sum_{j}\frac{C_{\mathrm{ij}}p_{j}}{\sum_{k}C_{\mathrm{kj}}f_{k}^{\mathrm{old}}}$

$U_{\mathrm{TV}}=\sum_{\mathrm{k. l}}\sqrt[{2}]{{\left(f_{k+1\mathrm{. l}}-f_{\mathrm{k.l}}\right)}^{2}+{\left(f_{\mathrm{k. l}+1}-f_{\mathrm{k.l}}\right)}^{2}+\varepsilon^{2}}$

 Where *p* is the projection data and $C_{\mathrm{ij}}$ is the contribution from image pixel i to projection bin j. β is a regularization parameter and β=0.001 was used $U_{\mathrm{TV}}$is the TV norm. The TV norm is used as an a priori regularization function to maintain the image edge and reduce noise. The number of iterations for ML-EM, CS-IR, and CS-IR with offset was fixed at 100, and a Butterworth filter (order, 8; cutoff frequency, 0.5 cycles/cm) was used as a pre-processing filter in reconstruction algorithms. We also used filtered back-projection (FBP) with a ramp filter and onset acquisition data (only 120 projections) as the reference image for evaluation criteria because FBP is an image reconstruction method that is theoretically correct and has excellent numerical stability. Neither scatter nor attenuation correction was performed for all reconstruction methods.

***Data analysis***

 The spatial resolutions were calculated as the full width at half maximum (FWHM) of the 2D (x- and y-axis) linear profiles in the transaxial images. The FWHMs obtained for the two axes were averaged. The image distortion was assessed by the aspect ratio (ASR) as

$\mathrm{ASR}=\frac{{\mathrm{FWHM}}_{\mathrm{radial}}}{{\mathrm{FWHM}}_{\mathrm{tangential}}}$

 Where *FWHM*_radial_ and *FWHM*_tangential _are the *FWHM* values in the radial and tangential directions, respectively. The linearity of the count density was examined by comparing the reconstructed SPECT counts and simulated values. Six circular regions of interest (ROIs) with areas of 80 mm^2^ were placed on the multi-cylinder parts and the mean SPECT counts were measured. We also investigated the properties of CS-IR with offset using the percent coefficient of variation (%CV) and specific binding ratio (SBR).

 Rectangle ROIs (900 mm^2^) were set on the 3D-

**Figure 3:**
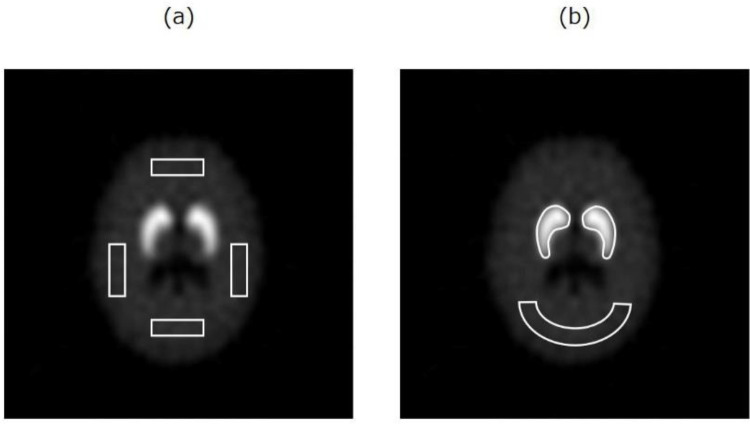
Region of interest settings for calculating the percent coefficient of variation (a) and specific binding ratio (b)

SDB phantom uniform area: top, bottom, left, and right (Figure 3a). The %CVs were calculated by

$\mathrm{\%CV}=\frac{{\mathrm{SD}}_{\mathrm{BG}}}{{\mathrm{MEAN}}_{\mathrm{BG}}}\times \mathrm{100}$

 Where *MEAN*_BG_ is the mean count in the uniform area ROIs and *SD*_BG_ is the standard deviation of the uniform area in the ROIs. The ROIs for striata were placed on the simulation images by manually contouring the striatal structure and were then copied to the reconstructed images. Similarly, an elliptical background ROI with an area of 2000 mm^2^ was placed in the occipital region of the phantom (Figure 3b). The SBRs were calculated as

$\mathrm{SBR}=\frac{{\mathrm{MEAN}}_{\mathrm{striatum}}-{\mathrm{MEAN}}_{\mathrm{occipital}}}{{\mathrm{MEAN}}_{\mathrm{occipital}}}$

 Where *MEAN*_striatum_ is the mean count in the striatal ROI and *MEAN*_occipital_ is the mean count in the background ROI.

***Statistical analysis***

 The linearity of the regression was analyzed by calculating the correlation coefficient between reconstructed SPECT counts and simulated values. The %CV obtained by each reconstruction method was compared using two-factor analysis of variance. Differences were considered statistically significant for P values less than 0.05.

## Results

***FWHM, ASR, and linearity***

**Figure 4:**
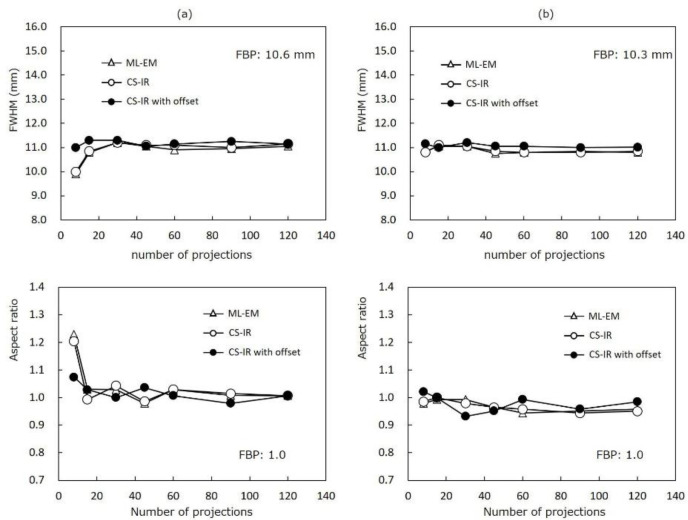
Graphs showing the full width half maximum (FWHM; upper row) and aspect ratio (ASR; lower row) for each reconstruction at (a) 10- and (b) 100-mm positions. The FWHMs obtained by compressed sensing (CS) offset acquisition were largely unchanged as the number of projections decreased. Although the FHWMs tended to be slightly higher by CS-IR with offset acquisition than by FBP, the ASRs obtained by CS-IR with offset acquisition were equal to 1.0 at all projection numbers

 The FWHMs and ASRs obtained by the ML-EM, CS-IR, and CS-IR with offset reconstruction methods are shown in Figure 4. In ML-EM and CS-IR, the FWHMs decreased when the number of projections was decreased from 30 projections or less. However, there was no clear change in the FWHM obtained by CS-IR with offset. The FWHMs at 10 mm for 120 projections were 10.6 mm by FBP, 11.1 mm by ML-EM, 11.2 mm by CS-IR, and 11.2 mm by CS-IR with offset. FWHMs at 100 mm for 120 projections were 10.3 mm by FBP, 10.8 mm by ML-EM, 10.9 mm by CS-IR, and 11.0 mm by CS-IR with offset. On the other hand, although the ASRs for each reconstruction remained equal to 1.0 as the number of projections decreased, clear increases in ASR were observed by ML-EM and CS-IR from 8 projections.

**Figure 5:**
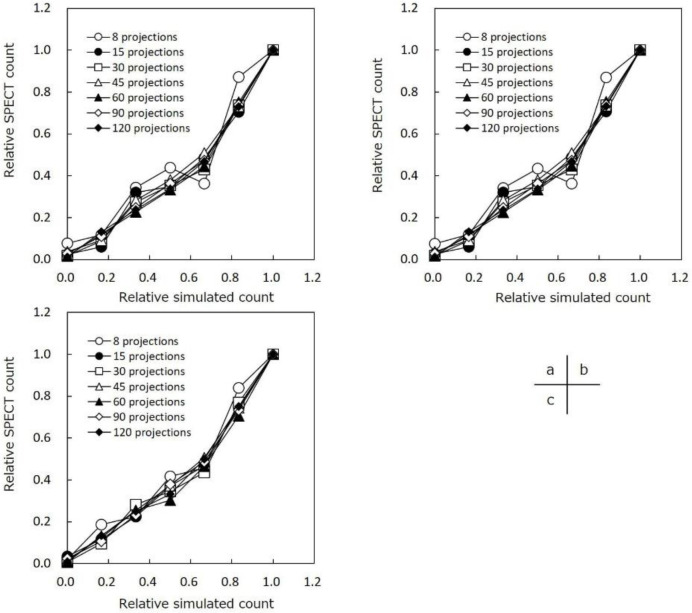
Correlation between the relative simulated count and single-photon emission computed tomography (SPECT) count: (a) maximum likelihood-expectation maximization (ML-EM), (b) compressed sensing iterative reconstruction (CS-IR), and (c) compressed sensing with offset acquisition. The correlation was good as the number of projections decreased. However, the linearity of the count density was not retained by ML-EM and CS-IR from 8 projections

Figure 5 shows the correlation between the reconstructed SPECT counts and simulated values obtained by ML-EM, CS-IR, and CS-IR with offset reconstruction methods. A significant correlation was observed between the reconstructed SPECT counts and simulated values from 15–120 projections. However, the linearity of the count density was not retained by ML-EM and CS-IR from 8 projections.

***%*** ***CV and SBR***

**Figure 6:**
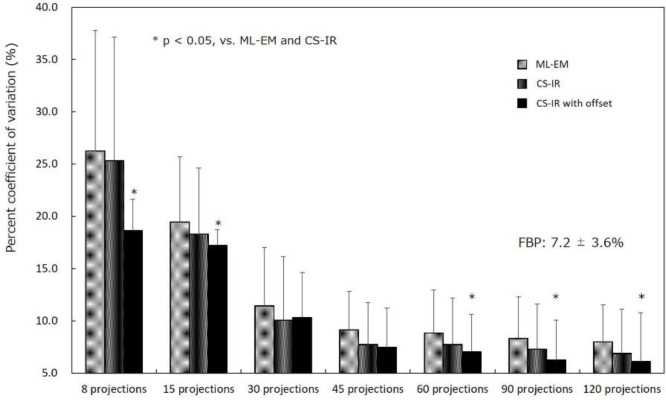
Percent coefficient of variation (%CV) as a function of the number of projections for each reconstruction. %CV values obtained by compressed sensing (CS) with offset acquisition were significantly lower than those obtained by the maximum likelihood-expectation maximization (ML-EM) and compressed sensing iterative reconstruction (CS-IR) methods. Similar %CVs were obtained by filtered back-projection FBP from 120 projections and by CS-IR with offset acquisition from 60 projections

 Comparisons of %CVs as a function of the number of projections obtained by each reconstruction method are shown in Figure 6. 

 Although the %CV increased as the number of projections decreased, the %CV obtained by CS-IR with offset (excluding 30 and 45 projections) was significantly lower than that obtained by ML-EM and CS-IR (p<0.05). The %CVs obtained from 120 projections were 7.2%±3.6% by FBP, 8.0%±3.6% by ML-EM, 6.9%±4.2% by CS-IR, and 6.1%±4.6% by CS-IR with offset. The %CVs obtained from 60 projections were 8.8%±4.1% by ML-EM, 7.7%±4.5% by CS-IR, and 7.0%±3.6% by CS-IR with offset. CS-IR with offset reduced the image noise level by 3.2%–26% compared with CS-IR. There was no significant difference between the %CV obtained from 60 projections by CS-IR with offset and that obtained from 120 projections by FBP.

**Figure 7:**
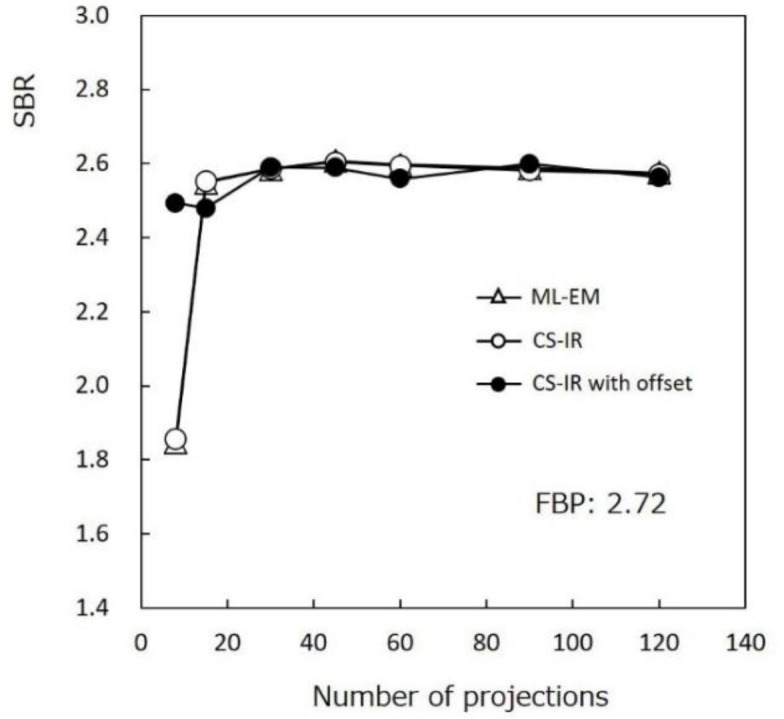
Speciﬁc binding ratio (SBR) comparisons between reconstruction methods. The SBR did not change with the number of projections by CS-IR with offset acquisition

Figure 7 shows the SBRs obtained by each reconstruction method. The SBRs from 120 projections were 2.72 by FBP, 2.57 by ML-EM, 2.57 by CS-IR, and 2.56 by CS-IR with offset. The SBRs tended to be slightly lower by ML-EM, CS-

IR, and CS-IR with offset than by FBP. However, SBRs showed minimal change for CS-IR with offset for different number of projections as some difference was observed.

**Figure 8:**
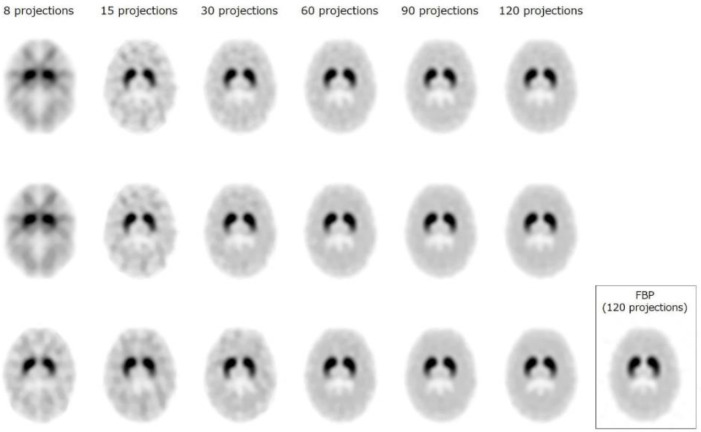
Reconstructed images of the striatal digital phantom using various numbers of projections obtained by maximum likelihood-expectation maximization (ML-EM, upper row), compressed sensing iterative reconstruction (CS-IR, middle row), and CS-IR with offset acquisition (lower row). Reference image: filtered back-projection (FBP) from 120 projections. CS-IR with offset acquisition reduced the image noise compared with other image reconstruction methods

 SPECT images of the 3D-SDB phantom are shown in Figure 8. Each image was normalized to its maximum count and is displayed using the same window level. Visual image quality obtained by CS-IR with offset from 60 projections was similar to that obtained by FBP from 120 projections. In addition, CS-IR with offset improved the image uniformity when compared with ML-EM and CS-IR. Although 

streak artifacts and TV-specific patchy patterns appeared in the undersampled CS recon-structed images (8–30 projections), these artifacts were mitigated by CS-IR with offset images. In particular, a sharp outline of the striatum and cerebral parenchyma was only maintained by CS-IR with offset from 8–15 projections.

## Discussion

 In image acquisition, the total acquisition time depends on the number of projections and the shoot acquisition time per projection. Because the total acquisition time affects the image quality and quantification, it is balanced by the need to acquire sufficient counts. One way to shorten acquisition times is to reduce the number of projections in a 360° acquisition. 

 Although this approach is simple, reducing the number of projections can decrease the image quality and increase the number and prominence of streak artifacts. Therefore, we assessed the effect of a combination of CS iterative reconstruction and offset acquisition from undersampled projection data. Our results indicated that CS-IR with offset improved the image quality without decreasing the quantification compared with FBP and CS-IR. In addition, it is possible that CS-IR with offset enables a greater reduction in the number of projections because a sharp outline of the striatal phantom is obtained from 8–15 projections.

 In previous studies, offset acquisition has enabled a reduction in the number of projections while maintaining the image quality for brain and myocardial SPECT (9, 14). In general, streak artifacts occur during back-projection in reconstruction (the same as in iterative reconstruction with forward/back-projection). Current SPECT acquisition methods with a constant angular interval (symmetric projection data) emphasize streak artifacts as the number of projections decreases because of the overlap of back-projected projections on a diagonal. The projection data obtained by offset acquisition were generated by offsetting opposite gamma cameras by half the step angle (asymmetric projection data). Therefore, offset acquisition reduced the streak artifacts and improved the image uniformity compared with current SPECT acquisition methods.

 To assess the spatial resolution and linearity of count density, we used the FWHM, ASR, and relative count density of the reconstructed images. Although the FWHMs and ASRs obtained by the ML-EM and CS-IR methods changed as the number of projections decreased, the FWHM and ASR obtained by CS-IR with offset remained constant. Furthermore, a significant correlation was observed between the reconstructed SPECT counts and simulated values by all reconstruction methods. However, the linearity of the count density for small projection numbers (8–15 projections) was only retained by CS-IR with offset. The optimal number of projections for SPECT images is defined by the sampling theorem (15). According to this theorem, the optimal number of projections was 111 in this study. Takahashi et al. reported that the spatial resolution obtained by an iterative recon-struction method from a smaller number of projections was similar to that obtained by other methods from a large number of projections (16). 

 In addition, we demonstrated that a larger number of projections is not essential for iterative reconstruction techniques, including CS iterative reconstruction (6). The combination of CS reconstruction and offset sampling provided the potential to reduce the number of projection data required compared with previous studies. Because streak artifacts depend on the structures of objects and might not occur for all objects, the number of projections by CS-IR with offset should be optimized for different clinical SPECT imaging approaches.

 %CVs increased as the number of projections decreased. However, CS-IR with offset decreased the image noise level compared with ML-EM and CS-IR. In addition, the %CV values obtained by CS-IR with offset from 60 projections were significantly lower than those obtained by FBP from 120 projections. In this study, a reduction in the number of projections implied a decrease in the total acquisition time and counts because the acquisition time per projection was kept stable. Our results indicate that offset acquisition is a good approach for undersampled SPECT imaging by CS iterative reconstruction, considering our previous findings. However, the effect of CS reconstruction was restricted in comparison with our previous study. The reason for this result is the β value setting. L1 regularization by TV strongly depends on the hyper-parameter β values. Although image uniformity is improved, a specific patchy pattern is visible in reconstructed images when the β values are higher. Therefore, we used β=0.001 (lower than in the previous study) because offset acquisition was expected to improve the image quality. Nevertheless, the combination of CS reconstruction with offset acquisition enabled a reduction in image noise with both regular and smaller projection numbers. β values are key to controlling the effect of the CS algorithm (smoothing level, low-contrast, and high-contrast). Further work is required to optimize the CS parameters for different SPECT imaging approaches.

 Although the SBRs obtained by CS-IR with offset were slightly lower than those obtained by FBP, our results showed that the values were similar. These results are also consistent with those obtained from smaller numbers of projections. Based on the results of linearity and image uniformity, offset acquisition seems to show a clear advantage for CS-based SPECT reconstruction regardless of the number of projections. However, the SBR values obtained by CS with offset acquisition were under-estimated. We did not perform resolution recovery because our aim was simply to evaluate the effect of CS-IR with offset. Further work is required to include the resolution recovery technique in CS with offset acquisition.

 In SPECT image reconstruction, it is clear that theoretically unrealistic reconstruction conditions from a small number of projections decrease the reconstructed image quality, especially by FBP. However, the striatum and brain detail was clearly visible by ML-EM and CS reconstruction under theoretically unrealistic reconstruction conditions. In particular, CS-IR with offset provided better reconstructed images compared with ML-EM and CS-IR from only 8 projections. This result raises the possibility of rapid dynamic SPECT acquisition using a commercially available dual-head SPECT camera. Our CS-IR with offset method can shorten overall SPECT acquisition times by drastically reducing the number of projections. Although validation of the proposed algorithm is necessary before its use with a variety of SPECT imaging techniques, the combination of CS reconstruction and offset acquisition will potentially be useful for studying dynamic perfusion or parametric imaging with SPECT. In this study, because we developed a challenging novel approach for rapid SPECT acquisition, offset acquisition is not implemented on current SPECT system. Although the image processing is needed to be implemented, it is possible that offset acquisition using continuous-mode acquisition with the small step angle.

 Our study has several main limitations. First, we performed only digital phantom research and our results might hold best when the acquisition count is sufficient. Image acquisition and reconstruction should be evaluated for several uptake patterns. Therefore, it is necessary to evaluate the effect of CS-IR with offset in clinical examinations using commercially available devices. Second, neither scatter nor attenuation was investigated in our simulation because the aim was simply to evaluate the effect of CS-IR with offset from undersampled projection data. Third, our simulation conditions were performed only one of the commercially available SPECT/CT device with collimator, and the Butterworth filter setting was fixed. Finally, resolution recovery improves the spatial resolution of SPECT images; thus, future studies should examine CS reconstruction including resolution recovery. 

## Conclusion

 We evaluated CS reconstruction combined with offset sampling acquisition from undersampled projections. Our algorithm provides good image quality and quantification from undersampled sparse-view SPECT projection data. A combination of CS reconstruction and offset sampling shortened the overall SPECT acquisition times, which would help to reduce motion artifacts and improve patient comfort in clinical SPECT imaging.

## Compliance with ethical standards

 This article does not contain any studies with human participants or animals.

## Conflict of interest

 The authors have no conflicts of interest to declare.
